# Impact of the applied simulated and integrated learning approach on nursing assistants’ knowledge and confidence caring for frail seniors in nursing homes

**DOI:** 10.1186/s40814-018-0272-x

**Published:** 2018-05-14

**Authors:** Veronique M. Boscart, George Heckman, Meaghan Davey, Michelle Heyer, John P. Hirdes

**Affiliations:** 1 CIHR/Schlegel Industrial Research Chair for Colleges in Seniors Care, Schlegel Centre for Advancing Seniors Care, Conestoga College Institute of Technology and Advanced Learning, Kitchener, Canada; 2 Schlegel Research Chair in Geriatric Medicine, Schlegel-University of Waterloo Research Institute for Aging, Waterloo, Canada; 3 Schlegel Centre for Advancing Seniors Care, Conestoga College Institute of Technology and Advanced Learning, Kitchener, Canada; 4 Conestoga College Institute of Technology and Advanced Learning, Kitchener, Canada; 5 School of Public Health and Health Systems, University of Waterloo, Waterloo, Canada

**Keywords:** Long-term care, Nursing homes, Comprehensive Geriatric Assessment, interRAI, Nursing assistants, Standardized assessment tools

## Abstract

**Background:**

Increasing importance is being placed on optimizing the role of Nursing Aides (NAs) in improving quality of care for nursing home (NH) residents. One approach to do so is to have NAs participate in assessments embedded within the Minimum Data Set (MDS). This pilot study aimed to design and evaluate the *Applied Simulated and Integrated Learning Approach (ASILA)* program, a novel innovative training program for NAs employed in NHs to enhance their ability to assess residents within an inter-professional framework.

**Methods:**

A mixed quantitative and qualitative repeated measures design was used to assess changes in NAs’ knowledge and perception of assessments and resident clinical outcomes. Additionally, focus groups were conducted with NAs upon completion of the ASILA program. A total of 23 NAs and nurses in NHs in two Canadian provinces participated. The ASILA pilot program consisted of three selected modules; each module including an evidence-informed case-scenario, assessments, the use of appropriate MDS tools and documentation, care planing and reporting systems. ASILA was delivered over the course of two days per home. The primary outcome measure focused on the impact of ASILA on NA knowledge and confidence in assessing residents and understanding the relevance and use of elements if the MDS tools. Secondary outcomes included NAs' satisfaction with ASILA and the impact of ASILA on resident clinical outcomes. Data were collected one week prior, immediately after, and three months after the ASILA program.

**Results:**

Following ASILA, NAs reported increased knowledge test scores and confidence in assessing residents by using MDS tools, although this did not reach significance after multiple testing (*p* = 0.0256 and *p* = 0.1541 respectively). NAs reported more confidence in providing care to residents (77.8%) and felt that the care provided was more resident-centered (83.3%) than before the ASILA program. There were no significant trends in improved resident outcomes following ASILA.

**Conclusion:**

Pilot findings indicate that the ASILA program could be a successful approach to support NAs to enhance their ability to assess residents in an inter-professional framework.

## Background

A rapidly increasing need to care for seniors requiring complex care highlights the need for skilled care in nursing home (NH) settings. It is estimated that by 2020, Canadian healthcare staff will spend 75% of their time with seniors requiring complex care [1]. The majority of these workers will be registered nursing staff and nursing aides [2].

Complex care is required for frail seniors, because their multiple deficits in multiple systems place them at increased risk of falls, disability, poor quality of life, institutionalization, and death [3, 4]. The evaluation of frail seniors is best operationalized with a Comprehensive Geriatric Assessment (CGA) [5]. A foundational requirement for effective CGA (i.e., comprehensive data collection and care planning) is appropriate geriatric training among healthcare staff. Training (entry-to-practice and continuing education) therefore must address the need for such expertise. However, insufficient geriatric content in healthcare education persists [6], leading to concerns that staff do not have adequate knowledge to assess and provide care [7, 8]. This deficit is especially acute in NH settings with negative consequences for seniors.

Difficulties in providing appropriate care to frail seniors are compounded by unfamiliarity with data and assessment systems used in the NH setting. interRAI instruments provide validated and reliable tools for a CGA and assemble data about a resident’s health, diagnoses, and function (i.e., cognition, communication and hearing, physical functioning, health conditions, and preventative health measures) [9, 11]. The Minimum Data Set (MDS), which was originally developed by interRAI, is mandated for baseline assessments and regular updates in most NH agencies in North America [10] (two of the researchers are interRAI fellows; however, they have no financial or general conflict of interest with interRAI). These instruments offer distinct advantages [11], including screening algorithms to identify those most likely to benefit from CGA, embedded scales and clinical action protocols to facilitate care planning, standardization and compatibility with electronic medical records, and care Quality Indicators and Case-Mix algorithms. Clinical trials of the newer interRAI Long Term Care Facility instrument demonstrated that these assessment systems improve NHs’ care quality [12].

Despite their widespread and mandated use, healthcare students are rarely exposed to interRAI instruments like the MDS during their training. As a result, NH staff are not only unskilled in gerontology, they are also unfamiliar with the use of these tools, leading to missed opportunities for care interventions and positive outcomes. In an attempt to close the loop on the delivery of high-quality care, all NHs require their staff to attend continuous education courses on specific geriatric topics (i.e., falls, delirium), as well as training or workshops on the use of interRAI instruments. Unfortunately, these courses are developed and delivered in isolation, impeding staff from effectively translating theoretical knowledge into actual practice.

This pilot study aimed to develop and evaluate the Applied Simulated and Integrated Learning Approach (ASILA), an educational model aimed at improving residents’ outcomes. ASILA was created by the co-investigators (VB and GH) and is based on the Ontario PSW Program Standards, the Association of Canadian Community College’s Nationals Educational Standards for Personal Care Providers, and the Ontario College of Nurses Entry-to-Practice Competencies and Practice Standards, and input from seniors and other knowledge users (i.e., families, patient advocates, resident councils, and educators) to prepare learners to better care for frail seniors with diverse needs. The primary objective of this study was to determine if ASILA raised NAs’ knowledge and confidence in conducting CGAs by using the MDS 2.0 to inform documentation and communication. The secondary objective was to determine if this pilot study affected resident outcomes over time.

## Methods

### Intervention

The pilot ASILA program included three interactive educational modules focused on assessing frail seniors with heart failure, accelerated functional decline, or expressive behaviors. Each module included a case study describing a scenario of a resident presenting with signs and symptoms; a video demonstrating the use of focused components of a CGA and MDS tools to assess, document, and communicate findings; and the NA’s role in the care team. All modules were pilot tested for effectiveness with five NAs, and refinements were made. ASILA was then delivered over 2 days per NH with support of a detailed training guide to ensure reliability across sites.

### Design and ethical considerations

The study employed a one group mixed repeated measures design and was approved by the University of Waterloo Research Ethics Board (UW-ORE 20512) and Conestoga College Research Ethics Board (CC-135). The principal investigator approached the NH directors to obtain approval for their staff to participate. Individual written consents for all study participants and NHs were obtained.

### Sample

Convenience selection based on geography and size was used to identify 10 NAs and 2 nurses per NH (*n* = 20), and there is no connection between the selected homes and the authors. Although the target population for the ASILA program was NAs, the study also included a limited number of nurses since they conduct CGAs and use MDS tools in collaboration with NAs. Both NHs were large (> 190 beds), part of a corporate chain, and located in semi-rural areas. NA participants selected up to 20 residents in their care. These sample numbers were sufficient to allow for the detection of a small pre- to post-difference in outcomes for a pilot feasibility study [13].

### Data sources and reported characteristics

To demonstrate feasibility of ASILA, data were collected from NAs, RNs, and one LPN and residents from April to July in 2015. NA and nurse questionnaires included demographics, satisfaction, MDS tool use, and geriatric assessment profiles [14, 15], adapted from the Geriatric Institutional Assessment Profile [14]. The Geriatric Assessment Profile included knowledge, professional issues, capacity for collaboration, resource availability, and institutional values and has undergone reliability and validity testing [15, 16]. NA and nurse questionnaires were developed and tested by authors. Questionnaires were collected at pre-, immediate post, and 3 months post-intervention. One semi-structured focus group took place per NH with all participating staff, immediate post-intervention, to explore the perceived impact and usefulness of ASILA in enhancing their ability to assess residents. For residents, the most recent MDS assessment at pre- and 3 months post-intervention was used to extract Cognitive Performance Scale (CPS), Activities of Daily Living (ADL), Depression Rating Scale (DRS), Changes in Health, End-Stage Disease, Signs, Symptoms (CHESS), and Pain Scale [17].

### Statistical analyses

Descriptive statistics described resident and staff demographics and staff questionnaires, including means and standard deviations for continuous variables and percentages for categorical variables. Comparative analyses for between-NH comparisons included independent *T* tests and Fisher’s exact tests. Repeated measures analysis of variance tests were conducted to analyze staff questionnaire domain score changes over time. All tests included a two-sided alpha of 0.05. Quantitative analyses were conducted using SAS software (9.4) (SAS Institute, NC).

Qualitative data underwent constant comparative analysis [18] and was reviewed separately by authors (NVivo 10). Thematic content emerging from the transcripts was organized into categorized concepts. Authors discussed findings based on consensus until data saturation was achieved [18]. An audit trail described all stages and decisions during the analysis.

## Results

Thirteen staff in NH-1 and ten staff from NH-2 participated. In total, 23 staff completed the baseline questionnaires, 22 (95.7%) staff completed the immediate post-program questionnaire, and 19 (82.6%) staff completed the 3-month questionnaires. Resident data was available for 30 residents (20 from NH-1, 10 from NH-2).

### Staff demographics

Participating staff consisted of 18 NAs, 4 registered nurses, and 1 registered licensed practical nurse (Table 1). The majority were female (78.3%), and the mean age was 41.1 ± 7.8 years. Fifteen staff had an NA certificate (69.2%), 6 a nursing diploma (26.1%), and the remainder had a university degree (12.9%). Average years of experience ranged from 8.5 to 11.9 (4.6–9.6 at the current NH) reflecting a similar profile to the overall staffing profiles NHs in North America [19, 20]. No significant differences were found between the NHs (*p* > 0.05).

**Table 1:** Staff demographics

Characteristic	All (%) *N* = 23	NH-1 (%) *N* = 13	NH-2 (%) *N* = 10	*p* value
Gender (female)	18 (78.3%)	10 (76.9%)	8 (80.0%)	0.85
Age	41.1 ± 7.8	41.1 ± 8.5	41.1 ± 6.9	0.94
Position				
Nursing assistant	18 (78.3%)	11 (84.6%)	7 (70.0%)	0.69
Registered nurse	4 (17.4%)	2(15.4%)	2 (20.0%)	
Registered licensed practical nurse	1 (4.3%)	0 (0.0%)	1 (10.0%)	
Schooling				
BScN	1 (4.3%)	0 (0.0%)	1 (10.0%)	0.65
Bachelor	1 (4.3%)	0 (0.0%)	1 (10.0%)	
Certificate	15 (65.2%)	9 (69.2%)	6 (60.0%)	
Diploma	6 (26.1%)	4 (30.8%)	2 (20.0%)	
Years of experience	10.0 ± 6.2	8.5 ± 4.4	11.9 ± 8.5	0.26
Years at facility	6.8 ± 3.3	4.6 ± 1.2	9.6 ± 6.0	0.06

### Staff’s ability to assess residents

Several areas of improvement in staff scores on assessment and knowledge of MDS tools to document and communicate findings was noted 3 months post-ASILA (Table 2). Scores for staffs’ “geriatric nursing knowledge/attitude” and “perceived resource availability” improved (*p* = 0.03 and *p* = 0.03 respectively). The “professional issues” domain did not appear to change over time; however, there may have been more perceived professional disagreements (*p* = 0.0004) and reduced perceived burden of upsetting behaviors (*p* = 0.02) following ASILA. The “capacity for collaboration” domain and “institutional values” domains did not change over time. There was no observed increase in MDS tool use following ASILA (*p* = 0.15).

**Table 2:** Staff member domains of change

Characteristic	Baseline test	Immediate post-test	3 months post-test	*β* estimate (standard error)	*p* value
Geriatric nursing knowledge/attitude score	132.0 ± 9.2	142.6 ± 10.6	139.3 ± 12.6	4.0 (1.68)	0.03
Professional issues					
Perception of care practices	23.4 ± 5.4	25.0 ± 4.9	23.3 ± 5.2	0.1 (0.70)	0.92
Staff disagreement	38.2 ± 8.8	38.2 ± 6.8	39.9 ± 9.6	0.1 (1.03)	0.93
Staff/family/patient disagreement	36.5 ± 6.0	37.8 ± 5.7	41.2 ± 6.7	2.0 (0.49)	0.0004
Staff satisfaction	15.0 ± 3.1	15.5 ± 2.3	14.7 ± 3.5	0.1 (0.17)	0.65
Perceived upsetting behaviors	10.1 ± 2.3	11.0 ± 3.2	10.9 ± 3.4	0.7 (0.36)	0.08
Burden of upsetting behaviors	17.9 ± 4.2	19.1 ± 4.0	9.8 ± 2.4	1.0 (0.41)	0.02
Capacity for collaboration	9.1 ± 2.2	9.1 ± 1.8	9.8 ± 2.4	0.3 (0.26)	0.26
Resource availability	24.4 ± 6.1	26.7 ± 6.0	26.7 ± 6.0	2.0 (0.90)	0.03
Institutional values regarding older adults and staff	25.5 ± 5.0	25.6 ± 4.5	26.7 ± 5.8	0.2 (0.40)	0.57
MDS instrument use	22.3 ± 4.2	23.9 ± 5.2	24.3 ± 6.9	1.2 (0.83)	0.15

### Staff satisfaction

Most staff perceived that, following ASILA, their role on the team changed (83.3%), and the care they provided was more resident-centered (83.3%) (Table 3). Staff felt more confident in providing care (77.8%), quality of teamwork, and communication improved (61.1%), and the team’s knowledge and skills increased (61.1%) after ASILA. Staff were less confident that the actual quality of care for residents improved after ASILA and that various resources were in place to assist with the implementation of ASILA.

**Table 3:** Staff satisfaction questionnaires

Questionnaire statement	% agreement
My role as a team member has changed.	15 (83.3%)
I am happy with my role on the team.	15 (83.3%)
I feel as though the care provided to the resident is more resident-centred.	15 (83.3%)
I feel more confident in providing care to residents.	14 (77.8%)
The quality of team work and communication has improved.	11 (61.1%)
The team’s knowledge and skills have improved.	11 (61.1%)
The quality of care residents have received has improved.	10 (55.6%)
The team’s attitudes have improved.	10 (55.6%)
Other staff have influenced the implementation of the ASILA program.	10 (55.6%)
Physical changes within the nursing home environment were required to institute the ASILA program.	10 (55.6%)
The presence of a clinical educator assisted with the implementation of the ASILA program.	10 (55.6%)
My previous work assisted with the implementation of the ASILA program.	10 (55.6%)
Improvements can be made to the implementation of the ASILA program.	10 (55.6%)
A cultural change was necessary to implement the ASILA program.	9 (56.3%)
Other resources have influenced the implementation of the ASILA program.	8 (50.1%)
The team’s practices at the facility have influenced the ASILA program.	7 (38.9%)
Managers and administrators have influenced the ASILA program	7 (38.9%)
The Director of the nursing home have assisted in the implementation of the ASILA program.	7 (38.9%)
The presence of a nursing home manager assisted with the implementation of the ASILA program.	6 (37.5%)
The presence of an Advisory Team assisted with the implementation of the ASILA program.	6 (37.5%)

### Staff perspectives of ASILA

Overall, staff believed ASILA to be very informative for their practice and they acquired new learning about MDS. Many staff stated that, before ASILA, they lacked a good understanding of the MDS instruments and a CGA as part of overall care planning. A NA stated, “I don’t think we ever knew how to actually read [MDS]. We were never shown in school, so that really changed. Being able to understand [MDS] when you’re looking at it. Because I would just see a bunch of numbers and try to guess what they meant, but now I know how it works” (NA1).

As a result of not understanding MDS assessments, several staff described not feeling comfortable using MDS tools. One NA said: “I don’t think this was ever meant for us to use [MDS]. The way they were set up in the charts, it didn’t really look like it was supposed to be accessible for us. They were never like, ‘hey, you can use this as a tool’… But if you actually look at [MDS], they are flow sheets. They are exactly our flow sheets that we fill out for each person” (NA9). The awareness that MDS is a comprehensive assessment and data tracking system supported participants in understanding how this information can be used for care planning.

Lastly, staff indicated that ASILA provided them with more confidence to recognize specific symptoms and reporting these to the team. An NA stated: “Being able to say these three symptoms, this is probably what it is. And being able to go confidently to a nurse and say: ‘Look, this is what’s happening. Let’s make sure we’re looking at proper diagnosis.’ Not letting it get to the point that legs are swollen halfway already” (NA3). Overall comments revealed a better understanding of the importance of CGAs and interpreting MDS to optimize quality care.

### Resident demographics

Residents’ mean age was 81.2 ± 14.5 years, with the majority being female (70.0%). The mean length of stay was 2.5 ± 2.1 years. Primary diagnoses included dementia (40.0%), depression (20.0%), hypothyroidism (20.0%), osteoporosis (20.0%), cerebrovascular accident (16.7%), arthritis (16.7%), congestive heart failure (13.3%), and anxiety (10.0%).

### Resident quality of care indicators

The indicators for residents are described in Table 4. The majority of residents presented with cognitive impairment before ASILA (90.0%), and these scores, as expected, increased to 93.3% 3 months later. Most residents were dependent as indicated by ADL scores (100.0% in NH-2 both pre- and post-ASILA, 75.0 and 80.0% respectively for NH-1). Several residents had mood symptoms as indicated by the DRS pre-intervention (71.4%), yet this decreased to 63.3% post-ASILA. The majority of residents showed some health instability as indicated by the CHESS scale both pre- and post-ASILA (56.7 and 60.0% respectively). A higher percentage of residents indicated to be in daily pain (16.7%) before ASILA as compared to post (10.0%).

**Table 4:** Resident characteristics

Characteristic	Baseline (all) *N* = 30	NH-1 *N* = 20	NH-2 *N* = 10	3 months post (all) *N* = 30	NH-1 *N* = 20	NH-2 *N* = 10
Age	81.2 ± 14.5	77.3 ± 16.1	89.2 ± 5.4	N/A	N/A	N/A
Gender (% female)	21 (70.0)	15 (71.4)	6 (60.0)	N/A	N/A	N/A
Length of stay (months)	30.4 (24.7)	34.0 (24.1)	23.1 (25.4)	N/A	N/A	N/A
Cognitive Performance Scale						
0 (intact)	3 (10.0)	2 (10.0)	1 (10.0)	2 (6.7)	2 (10.0)	0 (0.0)
≥ 1 (impaired)	27 (90.0)	18 (90.0)	9 (90.0)	28 (93.3)	18 (90.0)	10 (100.0)
Activities of Daily Living (short form—ON site only)						
0 (independence)	5 (25.0)	5 (25.0)		4 (20.0)	4 (20.0)	
≥ 1 (dependence)	15 (75.0)	15 (75.0)		16 (80.0)	16 (80.0)	
Activities of Daily Living (self-performance hierarchy—Alberta site only)						
0 (independence)	0 (0.0)		0 (0.0)	0 (0.0)		0 (0.0)
≥ 1 (dependence)	10 (100.0)		10 (100.0)	10 (100.0)		10 (100.0)
Depression Rating Scale						
0–2 (no disorders)	8 (28.6)	5 (25.0)	5 (50.0)	11 (36.7)	5 (25.0)	6 (60.0)
≥ 3 (disorder)	20 (71.4)	15 (75.0)	5 (50.0)	19 (63.3)	15 (75.0)	4 (40.0)
Changes in Health, End-Stage Disease, Signs, Symptoms Scale						
0 (not unstable)	13 (43.3)	9 (45.0)	4 (40.0)	12 (40.0)	8 (40.0)	4 (40.0)
≥1 (unstable)	17 (56.7)	11 (55.0)	6 (60.0)	18 (60.0)	12 (60.0)	6 (60.0)
Pain Scale						
0–1 (less than daily pain)	25 (83.3)	16 (80.0)	9 (90.0)	27 (90.0)	17 (60.0)	10 (100.0)
≥ 2 (daily pain)	5 (16.7)	4 (20.0)	1 (10.0)	3 (10.0)	3 (15.0)	0 (0.0)

*N/A* not applicable

## Discussion

The number of frail seniors requiring complex care in NHs is increasing [4]. Recognizing that comprehensive assessment and care planning systems are vital for responding to the strengths, preferences, and needs of this complex population, the uptake of interRAI assessments has been growing on a global basis. However, despite being the staff group providing most direct care to residents in NHs, NAs are not always valued for their contributions. This pilot study aimed to develop and test the Applied Simulated and Integrated Learning Approach (ASILA) program, an educational model for NAs, aimed to improve residents’ outcomes. The primary objective of this study was to determine if ASILA raised NAs’ knowledge and confidence in contributing to CGA by using MDS tools to inform documentation and communication. Findings indicate that 3 months post-ASILA, staff had an increased knowledge in the components of a CGA and MDS tools demonstrated in the video. Qualitative interviews indicated that staff valued the use of MDS in documentation and team communication, felt empowered to use MDS data to report changes in residents’ symptoms, and were more confident in providing care and that their care had become more resident-centered after ASILA.

Despite the positive qualitative findings of staff feeling they acquired new learning and awareness of using MDS tools, this pilot study did not observe an increase in MDS tool use. This could be the result of insufficient power, more time and support needed to learn to use MDS instruments, measurement tools that were not sensitive to capture long-term effects, or the need to refine the questionnaire to capture more meaningful outcomes. Additionally, as with any educational intervention, there are often obstacles in both participant behavior and knowledge, including staff relationships with residents, knowledge deficits, differences in care approaches, stigmas, and system barriers [21].

In terms of the second objective, resident outcomes, there appeared to be some change. Residents declined on cognition, ADL, and health instability; all occur frequently in a LTC environment. Functional decline because of multiple comorbidities is common in NH residents [22]. However, resident depression and pain scores showed some improvement; repeated mood symptoms on DRS decreased from 71.4% pre-intervention to 63.3% post-ASILA. For pain, the pre-intervention percentage of residents in pain was 16.7% pre-intervention and decreased to 10.0% post-ASILA. Both of these decreased scores may be related to improved care delivery. Given the small sample, limited observation period, multi-etiological nature of observed outcomes, and predictable decline of residents, some findings are unsurprising. Additionally, residents receive care from more than one NA. Yet, the increase in resident depression and pain scores may suggest that NAs paid more attention to assessment or were more confident in communicating their observations to the team. It would be interesting to conduct a larger study of longer duration to examine if clinical outcomes change.

To the authors’ knowledge, this was the first pilot study to deliver a tailored training program to NAs, focusing on a case study, outlining CGA, MDS tools, and the role of NAs on the care team. Staff data provided a rich description of the impact of ASILA on NA knowledge and role perceptions and how this translated to care practices. Knowledge scores and MDS use increased although this did not reach statistical significance. Further studies may find that increased staff knowledge and use of MDS results in meaningful changes in resident care and outcomes [9].

## Conclusion

This pilot study was conducted to develop and determine the potential effect of an educational program, which included case simulations and standardized assessment tools to support NAs in NHs. Staff knowledge and confidence using MDS tools increased. A larger multi-site study is needed to provide rigor evidence and may determine if the ASILA program could improve resident care and outcomes.

## Abbreviation

ASILA: Applied Simulated and Integrated Learning Approach
CGA: Comprehensive Geriatric Assessment
NA: Nursing aide
NH: Nursing home
